# Vaginal progesterone for prevention of preterm delivery in women with twin pregnancy: a randomized controlled trial

**Published:** 2018-06

**Authors:** OM Shabaan, IM Hassanin, AM Makhlouf, MN Salem, M Hussein, M Mohamed, AM Abbas

**Affiliations:** Department of Obstetrics and Gynecology, Faculty of Medicine, Assiut University, Assiut, Egypt; Department of Obstetrics and Gynecology, Faculty of Medicine, Sohag University, Sohag, Egypt; Department of Obstetrics and Gynecology, Manfalout General Hospital, Assiut, Egypt

**Keywords:** twins, progesterone, preterm labor, prevention, multiple pregnancies

## Abstract

**Background:**

The aim of this study is to evaluate the efficacy of vaginal progesterone to prevent preterm delivery in twin pregnancies and its effect on perinatal outcome.

**Materials and methods:**

A randomized, open label, controlled trial (NCT02350231) was carried out over 70 women, in three different hospitals in Egypt, between February 2015 and January 2017. All eligible pregnant women with twin pregnancies were randomly allocated in a 1:1 ratio into two groups. Group I (Progesterone group) was dispensed, 400 mg of progesterone through a vaginal pessary, each day at bedtime, from the 28th week of pregnancy until delivery. Group II (Control group) received no treatment other than the normal tonics taken during pregnancy. The two study groups were followed until delivery. The primary outcome was the rate of preterm delivery <37 weeks.

**Results:**

No significant differences were observed among both groups of women in terms of delivery <37 weeks (16.9% versus 25.4%; p=0.06) and mode of delivery (vaginal versus cesarean; p=0.31). The mean gestational age at delivery was comparable between both groups (p=0.09). Additionally, no difference, regarding the neonatal outcome, was observed between both groups.

**Conclusion:**

Dispensing vaginal progesterone [400 mg] after 28 weeks of gestation does not prevent preterm delivery in twin gestations.

## Introduction

Multiple pregnancies occur in 1–6 % of all births (Norman et al., 2009). More than 98% of these multiple births are twin births. The survival of preterm infants is directly related to the gestational age at delivery. Survival increases from less than 50% before 24 weeks to more than 95% by 33 weeks of gestation, plus, there is a corresponding inverse relationship between the risk of severe disability in survivors and gestational age at delivery (Rode et al., 2011). Several management strategies have been proposed to prevent preterm delivery in twin pregnancies including changing life style, medical treatment and even surgical interventions by cervical cerclage, however, none of them proved to be effective (Norman et al., 2009).

Antenatal progesterone supplementation has been proposed as a preventive measure for preterm birth in high risk singleton pregnancies, especially in patients with a short cervix and/or previous preterm delivery history (de Fonseca et al., 2003, Meis et al., 2003, Coomarasamy et al., 2006, Fonseca et al., 2007; Norman et al., 2009, Rode et al., 2011, Serra et al., 2013).

Several studies have investigated the preventive effect of progesterone on preterm birth in multiple gestations. Rode and colleagues (2011) reported that progesterone did not prevent preterm delivery in twin gestations and its administration did no harmful effects to the fetuses and infants after treatment. Likewise, Serra et al. (2013) reported that vaginal progesterone was in general well tolerated, but failed to prevent preterm births in dichorionic diamniotic twin pregnancies. Moreover, two aggregated data meta-analyses have examined the published trials of progestogens in twin pregnancies, but have not been sufficient. One of these did not differentiate between 17-hydroxyprogesterone caproate and vaginal progesterone (Likis et al., 2012). The other had too little information to investigate relevant subgroups (Sotiriadis et al., 2012).

A recent meta-analysis focused on women with a short cervix and found that vaginal progesterone reduced the rate of early preterm birth and the rate of composite neonatal morbidity/mortality in singleton pregnancies (Schuit et al., 2015).

Thus, given the small number of twins in that analysis, there was a trend towards the reduction of early preterm birth with progesterone and a significant reduction of neonatal morbidity/ mortality. None of the three previous meta-analyses of the effect of progestogens in twins included all published studies. Hence, although promising, exiting data on the use of progesterone to prevent preterm births in twins is not conclusive.

All the above studies and meta-analysis had been done on high risk of previous preterm birth or short cervix cases and none of them reported its role in low risk cases. Taking in consideration that twin pregnancy itself is a risk factor of preterm delivery; the current study aims to evaluate the efficacy of vaginal progesterone in prevention of preterm delivery in low risk twin gestation.

## Methods

### Study type, settings and duration

The current study is an open labeled, randomized, controlled trial (NCT02350231). The study was conducted in three tertiary care centers; Assiut University Women’s Health Hospital, Sohag University Hospital and Man out General Hospital, in Egypt, between February 2015 and January 2017. The Institutional Ethical Review Boards approved the study protocol. Before participation and after reading the patients’ information sheet or explicit lecture to the patient [if the patient was unable to read and/or write] to explain the nature of the study, all women signed an informed consent document.

### Study participants

All pregnant women with twin gestations who were invited to participate in this study. Pregnant women at 28 weeks of gestation presenting the following criteria: naturally conceived, dichorionic diamniotic twin gestation, uncomplicated pregnancy, no major fetal anomalies were included in this study. Women with a contraindication to progesterone treatment, higher multiple pregnancies, single fetal demise or fetal growth restriction of co-twin, polyhydramnios, threatened preterm labor, premature rupture of membranes, with a cervical cerclage from a previous or a current pregnancy, chronic medical diseases and difficulty for a regular follow-up were excluded.

### Sample size

Our sample size was calculated with a base on preterm delivery rate. Previous studies have demonstrated that the incidence of preterm delivery in twins is 50 % (Serra et al., 2013). Fonseca et al., (2007) showed that the use of vaginal progesterone in high-risk pregnancy decreased preterm delivery by 50 %. Thus, using a two-sided chi-square test with an α error of 0.05, a total sample size of 140 cases in the two groups (70 patients each) were required to demonstrate the difference between both groups with an 80% power (odds ratio=0.33) and a drop-out rate of 10% (Epi-info^TM^, CDC, USA. 2016).

### Randomization

Randomization was done using a computer- generated random table. Allocation concealment was done using serially numbered closed-opaque envelopes. Each envelope was labeled with a serial number and had a card noting the intervention type inside. One of the investigators had retained the randomization envelopes and did not share in patients counseling or care. When any of the centers had an eligible participant, she was assigned to either one of the study groups and did not change once assigned.

### Study intervention

At the time of recruitment, a detailed medical history (including personal, obstetric, menstrual and medical history) of all eligible participants was noted. Gestational age was estimated by a reliable date, and if not reliable, it was confirmed by a first trimester ultrasound crown-rump length. All pregnant women in the study received the usual antenatal care for twin pregnancy with iron and calcium supplementation. Moreover, all women were advised to rest through the entirety of the antenatal care program.

Eligible participants were randomly allocated in a 1:1 ratio to two groups. Group I (Progesterone group) received progesterone vaginal pessaries 400 mg (Prontogest® 400 suppositories; IBSA Pharmaceutical, Alexandria, Egypt) daily at bedtime from week 28 of pregnancy until delivery. Group II (Control group) women received no more treatment rather than the normal tonics taken during pregnancy. Adherence to treatment was ensured by asking participants to return empty packs at each visit.

### Follow-up

Women in both groups were followed up during their antenatal visits at the different centers every two weeks until delivery. At each visit they were asked for symptoms of preterm labor like heaviness, cramps, abdominal colic, and sudden gush of fluid. They were also subjected to ultrasonography scan to evaluate fetal wellbeing. Any complication occurring during the current pregnancy (placental abruption, intrauterine growth restriction (IUGR) and preeclampsia was recorded. The diagnosis of IUGR was based on three criteria: ultrasonographic deviation from the normal growth percentile, clinically detected suboptimal growth and birth weight inferior to the 10th percentile of the corresponding gestational age (Aban et al., 2004).

Moreover, participants were encouraged to deliver in the recruiting centers by providing each participant with a follow-up card to have her delivery for free in the designated facilities. Delivery data, any intrapartum or postpartum events, neonatal birth weight and referral to pediatric care unit (PCU) were also reported.

### Study outcomes

The primary outcome of this study was the rate of preterm delivery (before 37 weeks of gestation). Secondary outcomes included the occurrence of maternal complications as preeclampsia, the gestational age at delivery, neonatal birth weight at delivery, Apgar score at delivery, the perinatal mortality rate, the rate of admission to PCU, the rate of intraventricular hemorrhage, the rate of neonatal respiratory distress syndrome (RDS), need for mechanical ventilation, and the adverse effects of the study medication.

### Statistical analysis

Data entry and statistical analysis were done using SPSS software version 22 (Chicago, IL, USA). Qualitative variables were presented as frequency and percentage. Comparison between both groups was done by Chi-square test. Quantitative variables were presented in terms of mean and standard deviation and Student’s t-test was used for comparison between two groups. We considered a P value < 0.05 as significant.

## Results

**Figure 1:**
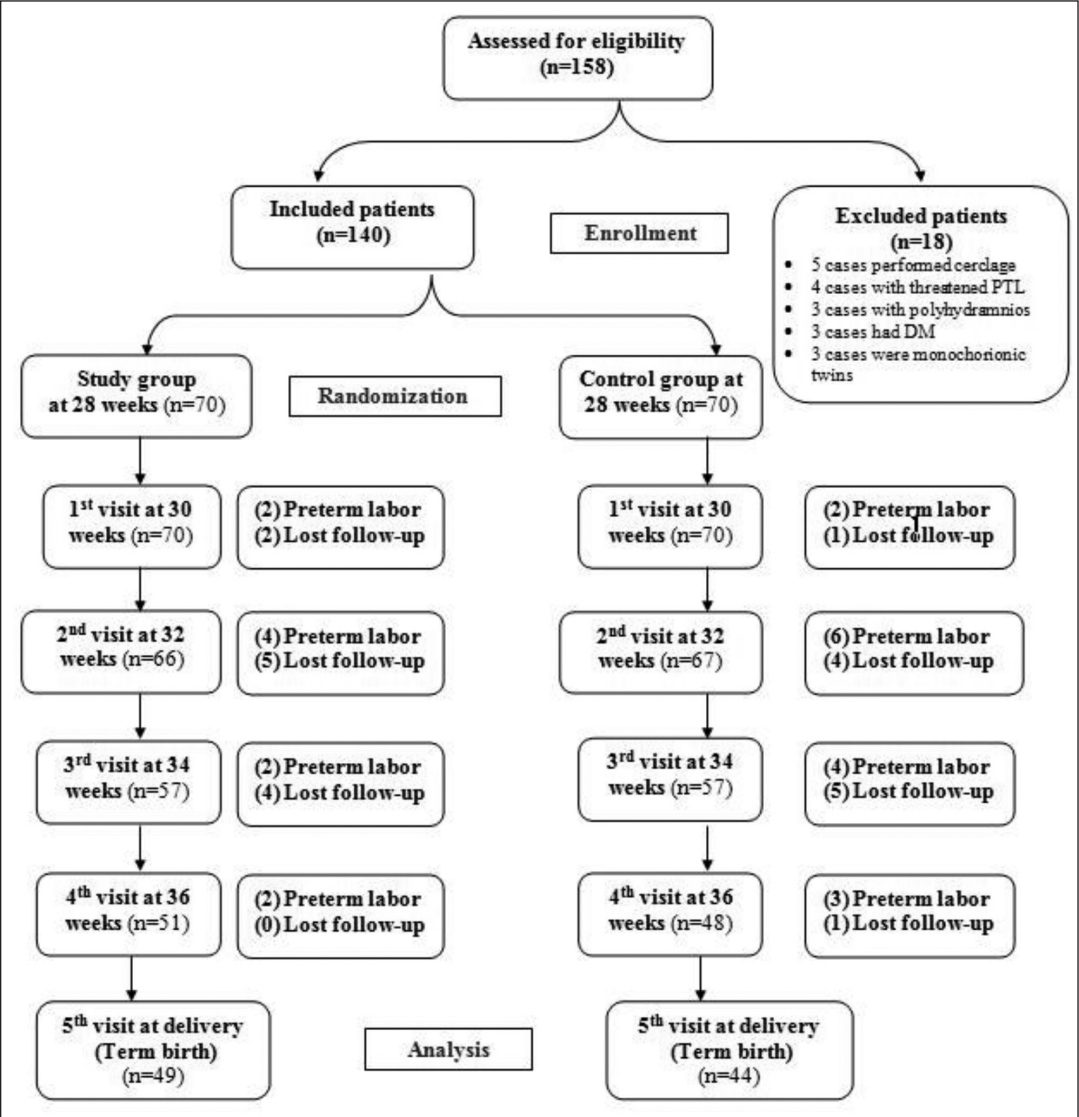
The study flowchart.

A total of 158 women were invited to participate in this study. Eighteen women were excluded because of not fulfilling eligibility criteria. We randomly assigned 140 twin-pregnant women at 28 weeks of their gestation were assigned into two groups (Figure 1). In each group, eleven patients failed to complete follow-up visits until delivery. Therefore, the final analysis included 59 women per each group.

Table I shows that both groups were comparable to baseline characteristics. The mean age of the study participants was ranged between 28.6-29.6 years and didn’t presented a significant difference between both groups. One-hundred eighteen babies were delivered in each group; 83.1% delivered at term in the progesterone group and 74.6% in the control group.

**Table I:** Baseline and clinical characteristics of the study participants at inclusion

Variables	Progesterone group (n= 70)	Control group (n= 70)	P-value
Age, years (mean±SD)	29.06 ± 4.06	28.61 ± 3.48	0.48
Consanguinity, n (%)	19 (26.4)	21 (29.2)	0.71
Parity, (mean±SD)	2.03 ± 1.69	2.14 ± 1.57	0.61
Previous miscarriage, n (%)	10 (13.9)	8 (11.1)	0.61
Gestational age by US, weeks (mean±SD)			
First fetus	28.9 ± 0.6	28.9 ± 0.3	0.95
Second fetus	28.8 ± 0.7	28.9 ± 0.4	0.32
Estimated fetal weight by US, grams (mean±SD)			
First fetus	1122.8 ± 201.4	1113.4 ± 140.1	0.78
Second fetus	1103 ± 187.2	1110.6 ± 122.1	0.82

SD = standard deviation, US = ultrasound

Regarding the primary outcome, 10 women (16.9%) in progesterone group versus 15 women (25.4%) in the control group delivered before the 37th week with no significant difference between both groups (p=0.06). As regard to pregnancy complications, the number of women who developed preeclampsia was not statistically different between the two groups 3/59 (5.1%) in the progesterone group versus 1/59 (1.7%) in the control group. In the progesterone group, only 4 patients developed IUGR versus 3 patients in the control group. None of the women in either group developed a placental abruption. Additionally, about half of the pregnancies were delivered by CS with no statistical difference regarding the mode of delivery between both groups (p=0.31) (Table II).

**Table II:** The study outcomes in both study groups

Outcomes	Progesterone group (n= 59)	Control group (n= 59)	P-value
Preterm birth <37 weeks	10 (16.9)	15 (25.4)	0.06
Term live birth	49 (83.1)	44 (74.6)	0.13
Preeclampsia	3 (5.1)	1 (1.7)	0.62
IUGR	4 (6.8)	3 (5.1)	0.69
Mode of delivery			
Vaginal	31 (52.5)	26 (44.1)	0.31
Cesarean	28 (47.5)	33 (45.9)	

IUGR = intrauterine growth restriction. All data are presented as n (%).

Table III shows the delivery data in both groups. The mean gestational age at delivery was comparable between both groups (p=0.09). Both groups were similar for mean weight at birth of both babies. Moreover, no differences regarding the Apgar score at 1 min < 7/10 (p=0.31), incidence of neonatal RDS (p=0.18), intraventricular hemorrhage (p=0.63), need for mechanical ventilation (p=0.37), admission to PCU (p=0.07) and perinatal mortality rate (p=1.0) were observed between neonates in both groups.

**Table III:** The neonatal outcomes at delivery of the study groups

Variables	Progesterone group (n= 118) ^a^	Control group (n= 118) ^a^	P-value
Gestational age, weeks	37.8 ± 1.5	37.0 ± 2.2	0.09
Birth weight, grams			
First baby	2723.6 ± 317.9	2608.4 ± 530.8	0.12
Second baby	2732.6 ± 337.8	2607.3 ± 493.5	0.08
Apgar score at 1 min <7/10	29 (24.58)	35 (29.66)	0.31
Respiratory distress syndrome	20 (16.95)	27 (22.88)	0.18
Intraventricular Hemorrhage	2 (1.69)	3 (2.54)	0.63
Admission to pediatric care unit	29 (24.58)	45 (38.13)	0.07
Need for mechanical ventilation	11 (9.32)	16 (13.56)	0.37
Perinatal mortality rate	1 (0.8)	1 (0.8)	1.0

All data are presented as n (%) or mean± standard deviation ^a^ The number of neonates in each group is higher than the number of women because there were 2 neonates in each pregnancy

No major adverse effects from the medication were reported among participants in this study. Eleven out of 59 (25.4%) women in the progesterone group complained from vaginal burning sensation and excessive vaginal discharge. Only two of them were diagnosed for bacterial vaginosis (BV) and were treated accordingly. in the control group, four patients (6.8%) complained for the same symptoms, three were diagnosed with BV.

## Discussion

In this multicenter-randomized open-labeled study, we demonstrated that administration of daily 400 mg vaginal progesterone starting at 28 weeks of gestation in diamniotic twin pregnancies failed to decrease the rate of preterm delivery with no reduction of perinatal morbidity or mortality.

Several trials have demonstrated the potential beneficial effect of progesterone supplementation to prevent preterm labor (PTL) most pertained to singleton pregnancy (Al revic et al., 2013) and only few studied multiple pregnancies (Brizot et al., 2015, El-refaie et al., 2016). In our study, we chose vaginal progesterone rather than oral or intramuscular synthetic progesterone because of its convenience for the patient. It is easy to use and is assumed to have a better bioavailability compared to intramuscular injections, and no major undesirable side effects, as occur when orally administrated [e.g. sleepiness, fatigue and headache] or side effects from intramuscular use [injection site pain, itching and swelling] (Maher et al., 2013).

The mean gestational age at delivery was 37.8 and 37.0 weeks in both, the progesterone and control groups, respectively (p=0.09). Similarly, birth weight at delivery in the progesterone group was (2723.6 and 2732.6 grams) for the first and the second baby, respectively, and in the control group (2608.4 and 2607.3 grams) for the first and second baby respectively with no statistical significant differences between both groups.

The presented results are in line with those from Brizot et al. (2015) in which 200 mg of daily vaginal progesterone was administered to women with twin pregnancies between the 18th and 21st week and 6 days of gestation till 34 weeks and 6 days in Brazil. This Brazilian study included 189 women in a progesterone group and 191 in a the placebo group for final analysis. No difference (p=0.095) in the mean gestational age at delivery was observed between progesterone (35.08±3.19 weeks) and placebo groups (35.55±2.85 weeks). The incidence of spontaneous delivery at <34 weeks’ gestation was 18.5% in the progesterone group and 14.6% in the placebo group. No difference in the composite neonatal morbidity and mortality were observed between both groups (progesterone (15.5%) versus placebo (15.9%).

Our results also agreed with the Spanish RCT (Randomized Controlled trial) conducted by Serra and colleagues in 2013. In this study, dichorionicdiamniotic twin pregnancies used two daily vaginal pessaries, containing placebo, 200 mg or 400 mg of natural progesterone from 20 to 34 weeks of gestation or delivery (Serra et al., 2013). The Spanish group showed similar pregnancy and neonatal outcomes between the progesterone and the placebo groups. The proportion of preterm and very preterm births, low birth weight, perinatal mortality and neonatal morbidity showed no differences between the three groups. The 400-mg progesterone dose offered no advantages over the 200-mg regimen.

Additionally, the current study agreed with the meta-analysis of thirteen trials (3768 women) conducted by Schuit et al., 2015 which tested the effectiveness of progestogen treatment in the prevention of PTL in twin pregnancies (Schuit et al., 2015). Neither 17-hydroxyprogesterone caproate nor vaginal progesterone reduced the incidence of adverse perinatal outcomes (RR=1.1; 95% CI 0.97–1.4 and RR=0.97; 95% CI 0.77–1.2 respectively). This meta-analysis demonstrated a beneficial effect of progesterone dinistration in the subgroup of women with a cervical length ≤ 25 mm at randomization.

On the contrary, an Egyptian study carried out by El-refaie et al. (2016) including 250 women with dichorionic twin pregnancy, reported significantly lower neonatal morbidities and mortality by a lower incidence of very low 1500 gm. In this study, women were divided in two groups. One study group (n =125) receiving a daily dose of 400 mg of vaginal progesterone in suppositories at 20–24 weeks of gestation, and a second, the control group (n =125) received no treatment. The duration of pregnancy was significantly longer in the study group and the incidence of preterm labor before 34 and 32 weeks of gestation was significantly lower in the study group. The neonatal morbidities and mortality were significantly lower in the study group as shown by lower incidence of very low 1500 gm, birth weight, neonatal RDS, the need for mechanical ventilation and early neonatal death.

Our study does not recommend the general use of vaginal progesterone to prevent preterm labor of twin pregnancy regardless of the cervical length. Bias was avoided by central randomization and allocation concealment. However, some limitations could not be avoided. These included, patient blinding (placebo) and sample size, calculated for a 50% reduction in PTL with progesterone treatment as smaller, though important, differences could not be detected by our sample size. Furthermore, the sample size did not allow subgroup analysis according to cervical length or other specific risk factors.

In conclusion, a daily administration of 400 mg of vaginal progesterone after 28 weeks of gestation does not prevent the occurrence of preterm delivery in twin gestations and does not reduce perinatal morbidity and mortality.

## Conflicts of interest:

The authors declare that they have no conflict of interest.

Funding: No funding is provided for the study.
